# Analysis of In Vitro Effects of Sex Steroids on Lymphocyte Responsiveness in Murrah Buffaloes (*Bubalus bubalis*)

**DOI:** 10.1155/2012/139589

**Published:** 2012-04-29

**Authors:** Zahoor Ahmad Pampori, Sujata Pandita

**Affiliations:** Dairy Cattle Physiology Division, National Dairy Research Institute (ICAR), Haryana, Karnal 132001, India

## Abstract

Present study was carried out on forty four apparently healthy Murrah buffaloes of different age groups of both sexes to investigate the effects of sex steroids on cell mediated immunity in vitro. Estrogen inhibited proliferation in mitogen-stimulated lymphocytes from prepubertal but not post pubertal buffaloes of either sex. Estrogen at 100 pg/mL concentration stimulating the proliferation significantly (*P* < 0.05). in all groups and had higher stimulatory effect in lymphocytes from day 10 than day 0 of estrous cycle. Progesterone inhibited lymphocyte proliferation, and inhibition was directly related to the dose, in all groups of either sex. Testosterone did not inhibit proliferation at any dose level and did not show any consistent and lucid effects on lymphocyte proliferation. Present study revealed that buffalo lymphocytes produce appreciable amounts of NO in culture system after treatment with estradiol. Significantly high levels of NO in culture supernatant were found in prepubertal buffalo calves and least in post pubertal buffaloes, which had an inverse relation with lymphocyte proliferation in presence of estradiol. NO in culture supernatant was high at the lowest dose of progesterone which was proportional to the lymphocyte proliferation when treated with progesterone. No significant difference in NO culture supernatant was observed between different concentrations of testosterone treatment.

## 1. Introduction

The sex steroids are in use for the last more than a millennium to correct reproductive disorders and augment reproductive efficiency in animals. However, it has been increasingly apparent that the effects of sex hormones extend far beyond their predominant role in sexual differentiation and reproduction and are now being realised as integral signalling modulators of mammalian immune system. Sexual dimorphism in disease incidence and host response in wide range of animal species provides an indirect evidence of role of sexual hormones in immune modulation [1–3]. Immunological evidence suggests that female sex hormones play a role in the aetiology and course of chronic inflammatory diseases [4]. The number and activity of immune cells in reproductive tract of animals and humans vary significantly throughout the phases of the reproductive cycle and are believed to be controlled by changes in the levels of estradiol and progesterone hormones [5–7]. Ramadan et al. [8] in sheep and Wulster-Radcliffe et al. [9] in gilts have reported higher resistance to uterine infections at oestrus and least during luteal phase. Lymphocyte proliferation response, a promising marker of host immunity, has been reported to exhibit variability during various physiological and reproductive phases in different animal species ([10, 11] in dairy cattle; [12] in hamsters) that has been attributed to sex hormones. Use of sex steroids or sex steroid blockers or ovariectomy or castration in animal models for studying the pathophysiology and immunoendocrinology has provided direct evidence for the role of sex steroids in immune modulation [13, 14]. Since there existed species variation in immune responses to sex steroids, present study was taken up in buffaloes of different age, sex, and reproductive phase wherein phytohemagglutinin- (PHA-) stimulated lymphocytes were exposed to different concentrations of estrogen, progesterone, and testosterone to analyse the influence of these steroids on proliferation response and nitric oxide production, which probably is the first study of its kind in buffaloes.

## 2. Materials and Methods

Forty-four apparently healthy Murrah buffaloes of three age groups of both sexes (male and female) were selected from NDRI herd for conducting present experiments. Eighteen male buffaloes were divided into 3 groups of 6 buffaloes on the basis of their age. Group-I included buffaloes with age ranging between 11 and 13 months; group-II consisted of buffaloes with age ranging between 22 and 24 months. Group-III had postpubertal buffaloes 34 and 36 months of age. Similarly 18 female buffaloes were grouped exactly the same way as in males except that female post pubertal group had cyclic heifers at day 8–10 of estrous cycle. Eight cyclic heifers were selected and studied on day 0 and day 10 of estrous cycle. The estrum (day 0) was confirmed through rectal palpation by an expert and concerned veterinarian at the farm. All these Murrah buffaloes were maintained under routine management and nutritional practices as followed in the herd at the institute. The animal experiments were acceptable to the ethical standards of the National Dairy Research Institute, Karnal, India vide IAEC no. 23/09-21/11.

The lymphocytes from peripheral blood were isolated as per the method followed by Young et al. [15] with some alterations made by Huozha et al. [16] to suit our requirements. The proliferative response of lymphocytes was analysed using the colorimetric MTT (tetrazolium) assay, as described by [17]. Briefly, about 15 mL blood was drawn from every animal in heparinised vacutainer tubes from jugular vein, in the morning. Plasma was separated by centrifugation in refrigeration centrifuge at 2000 rpm for 30 minutes. A white buffy coat at the top of red column was harvested in a new sterile polypropylene centrifuge tube (15 mL) containing Dulbecco's phosphate buffered saline, (DPBS) in a ratio of 1 : 2 V/V and the whole contents were carefully layered over a lymphocyte separation medium (Histopaque-1077) in a ratio of 3 : 1 v/v in another sterile centrifuge tube and centrifuged at 1500 rpm for 40 min at room temperature to separate the lymphocytes. The lymphocytes in a thin, white layer at the interface were carefully pipetted out into next sterile tube containing 7 mL DPBS with antibiotics. Lymphocytes were washed twice in DPBS and third time in culture medium by refrigeration centrifugation at 1000 rpm for 10 minutes in each wash. After last wash culture medium was discarded and cells were resuspended in 3 mL culture medium (RPMI 1640 with 10% FCS and antibiotics).

Trypan blue dye exclusion method was used to determine the proportion of viable cells in the separated lymphocytes. When the lymphocyte viability was above 95%, the lymphocytes were processed further for culture. Culture of lymphocytes was carried out in 96-well, tissue culture, flat bottom, sterile microplates with lid from Greiner Bio-One, Germany. The cells were adjusted to 1 × 10^6^ cells per culture well. The culture medium used in present study was RPMI-1640 from Sigma Chemical Co., St. Louis, MI, USA, supplemented with L-glutamine, sodium bicarbonate, sodium pyruvate, and antibiotics (Penicillin G and streptomycin sulfate and antifungal amphotericin), besides 10% fetal calf serum. 20 *μ*L of phytohemagglutinin (PHA-P) was added per well to yield a concentration of 5 *μ*g/mL of culture medium. 20 *μ*L of steroid hormone (working concentration) was added to each well. The final volume in each well after adding mitogen and or sex steroid hormone was made to 200 *μ*L with culture medium. Blank wells contained only 200 *μ*L of culture medium with no cells, whereas S–M and S + M wells contained no mitogen and mitogen, respectively, besides lymphocytes. Every observation was made in triplicate wells. The cells were cultured for 36 hours at 37°C in a humidified CO_2_ incubator (5% CO_2_). After 36 hours of incubation, 100 *μ*L of culture supernatant was harvested in sterile 1.5 mL eppendorf tubes and stored at −20°C for estimation of nitric oxide. Equal volume of culture media with supplements, preincubated at 37°C, was added into the wells. 20 *μ*L of the MTT solution (5 mg/mL dissolved in DPBS and filtered through 0.22 *μ*m Millex-GV filter unit) was added to each well. The plates were again incubated for 4 hr at 37°C in humidified CO_2_ incubator. Thereafter, the supernatant was pipetted out completely without disturbing formazan crystal layer and 150 *μ*L of dimethyl sulfoxide (DMSO) was added to each well. After incubating the plate at room temperature for 15 minutes, it was shaken on a microplate shaker and the optical density was read using ELISA reader (Microscan MS-5608A) in dual wavelength measuring system, at a wavelength of 570 nm and a reference wavelength of 630 nm. Lymphocyte blastogenic response was expressed as Proliferation Index (PI) and was calculated as follows:

(1) $$\begin{array}{cc} \text{Proliferation}\;\text{Index}\;\left(\text{PI}\right) & =\text{OD}\;\text{of}\;\text{the}\;\text{mitogen}\;\\ & \;\;\text{stimulated}\;\text{cells}/\text{OD}\;\text{of}\;\\ & \;\;\text{the}\;\text{unstimulated}\;\text{cells}. \end{array}$$

Influence of steroid hormone on lymphocyte blastogenic activity was calculated by dividing optical density of hormone-treated cells with optical density of mitogen-stimulated cells.

Phytohemagglutinin used as a mitogen for proliferation of T-lymphocytes was made in culture medium at 50 *μ*g/mL. 10 *μ*L of it was used for every 90 *μ*L culture medium in each culture well to yield concentration of PHA 5 *μ*g/mL. Stock solution of estradiol, progesterone, and testosterone was prepared in ethanol which was diluted with culture medium to working concentrations of 10 ng/mL, 5 ng/mL, 1 ng/mL, and 100 pg/mL for estrogen, 10 *μ*g, 5 *μ*g, 1 *μ*g, and 100 ng for progesterone, and 1000 ng, 100 ng, 10 ng, and 1 ng/mL for testosterone. 10 *μ*L of working steroid solutions was added to every 90 *μ*L culture medium in culture well yielding final concentration of steroids as 1 ng/mL, 500 pg/mL, 100 pg/mL, and 10 pg/mL for estrogen, 1000 ng/mL, 500 ng/mL, 100 ng/mL and 10 ng/mL for progesterone, and 100 ng/mL, 10 ng/mL, 1 ng/mL, and 0.1 ng/mL for testosterone. All the steroid hormones, *β*-Estradiol, Progesterone (Δ^4^-pregnen-3-20-dione) and Testosterone (Δ^4^-androsten-17*β*-ol-3 one), Histopaque 1077, Phytohemagglutinin (PHA-P), Trypan blue, and Thiazolyl Blue Tetrazolium Bromide (MTT) were purchased from Sigma Chemical Co., St. Louis, MI, USA. Dulbecco's Phosphate Buffered Saline (DPBS) was purchased from Himedia Laboratories Pvt. Ltd. India. 

Plasma nitric oxide was estimated as total nitrite (NO_*x*_) using modified Griess reaction as described by Miranda et al. [18]. The test involved preparation of Griess-I (1% sulphanilamide w/v in 5% HCL) and Griess-II (0.1% N-I-naphthyl ethylenediamine dihydrochloride w/v in milli-Q water). The reagents were made fresh and filtered before use. The chemicals were purchased from Sigma Chemical Co., St. Louis, MI, USA. 50 *μ*L of test culture supernatant and standards (sodium nitrite at 1.56, 3.12, 6.25, 12.5, 25, 50, 100 *μ*M) prepared in culture medium was pipetted out into a 96-well microtitre plate and blank wells contained culture media only. The tests, blanks, and standards were run in triplicates. 50 *μ*L Griess I (1% sulphanilamide) was added to each well and held at room temperature for 5 minutes. 50 *μ*L Griess II was then added to each well and plates were incubated at 37°C for 30 min. After incubation, the absorbance was read at 540 nm wavelength in ELISA plate reader (Microscan MS-5608A). The concentration was calculated from the standard curve using linear regression equation. Detection limit of NO was 1.56 *μ*M. Inter-assay and intra assay difference was 3.4% and 4.62%, respectively.

The data analysis was performed using Systat 12 software package 2007 (Systat Software Inc. 1735 Technology Dr., Ste. 430, San Jose, CA 95110, USA). Analysis of variance of the data was performed using three-way ANOVA with variables age, sex, and concentration included in the model as fixed effects and Tukey's honestly significant difference test was employed. Values are presented as mean ± S.E. Graphs and charts were prepared in Microsoft Excel 2007.

## 3. Results

### 3.1. Effects of Sex Steroids on Lymphocyte Proliferation

The effect of four different levels of estradiol (10, 100, 500, and 1000 pg/mL**)** on lymphocyte proliferation response that was calculated as proliferation index (PI), in buffalo lymphocytes cultured in vitro, is presented in Table 1. Proliferation response, irrespective of concentration of estradiol added, was inhibited by 3 and 1%, respectively, in lymphocytes from both male (0.97 ± 0.01) and female (0.99 ± 0.01) buffaloes that did not differ significantly. However, significant (*P* < 0.001) variation between age groups and treatment levels was observed in the present study. Estrogen was found inhibiting proliferation in group-I and group-II buffaloes by 5% as compared to group-III post pubertal buffaloes where it stimulated the proliferation to the level of 4%. Among all the groups, post pubertal female buffaloes registered the highest (*P* < 0.05) PI (1.09 ± 0.03) with estrogen treatment. Among the four concentrations of estrogen, 100 pg/mL registered significant (*P* < 0.001) stimulatory effect on PI (1.05 ± 0.02) to the level of 5% as compared to 1000 pg/mL (0.93 ± 0.02) and 10 pg/mL (0.95 ± 0.01) that instead inhibited lymphocyte proliferation by 7% and 5% respectively.

Estrogen treatment of the lymphocytes collected from cyclic buffaloes on day 10 registered significantly (*P* < 0.05) higher stimulatory effect on PI to as high as 10% (1.10 ± 0.03) as compared to 3% (1.03 ± 0.01) on day 0 of estrous cycle. Among the estrogen concentrations, 1000, 500, and 100 pg/mL stimulated the lymphocyte proliferation (1.12, 1.10, and 1.07) significantly (*P* < 0.05) to the level of 12%, 10%, and 7%, respectively, whereas low level (10 pg/mL) inhibited proliferation (0.99 ± 0.03) up to 1% in cyclic buffaloes. Thus, in cycling females only low level of estrogen was found to be inhibitory to lymphocyte proliferation while all other concentrations were stimulatory.

Effects of progesterone treatment on in vitro proliferation of buffalo lymphocytes registered in present study are presented in Table 2.

Though progesterone inhibited lymphocyte proliferation significantly (*P* < 0.01) in both males and females, the inhibitory effect was severe in males (0.90 ± 0.01) to the level of 10% as compared to females (0.97 ± 0.01) only 3%. There was no significant difference in the effects of progesterone between different age groups of buffaloes. Progesterone inhibitory effects were significantly (*P* < 0.001) dose-dependent and PI was inversely proportional to the progesterone concentration. The highest concentration of progesterone (1000 ng/mL) inhibited proliferation (0.81 ± 0.02) by 19% whereas the lowest (10 ng/mL) registered stimulatory effect (1.058 ± 0.02) of 6%. Among all groups, group-III post pubertal female buffaloes registered highest (*P* < 0.01) proliferation (1.065 ± 0.02) and group-III post pubertal males the lowest (0.86 ± 0.03) when treated with progesterone.

The progesterone effects were not significantly different between the lymphocytes from the day 0 and day 10 of reproductive cycle. However, dose dependent effect of progesterone on proliferation was significant (*P* < 0.01) with highest concentration 1000 ng/mL progesterone inhibiting proliferation (0.976 ± 0.02) by 2% and 10 ng/mL stimulated proliferation (1.103 ± 0.02) by 10%. The trend in lymphocyte proliferation with decreased dose of progesterone was linear.

The effects of testosterone treatment on in vitro proliferation of buffalo lymphocytes of different sex and age are presented in Table 3. Proliferation index was not found to be different in the two sexes in response to testosterone supplementation in the culture. Males and females registered stimulation in proliferation (1.026 ± 0.01 and 1.05 ± 0.01) to the level of 2 and 5%, respectively. Testosterone in different concentration did not affect the lymphocyte proliferation significantly. The highest concentration of 100 ng/mL neither stimulated nor inhibited proliferation (1.00 ± 0.02). Other treatments of 10, 1, and 0.1 ng/mL concentration did stimulate proliferation (1.05 ± 0.02, 1.052 ± 0.02, and 1.048 ± 0.02) equally to the level of 5% which was statistically nonsignificant between the concentrations. Testosterone significantly (*P* < 0.01) inhibited proliferation by 4% in group-II males but stimulated by 13% in group-I males, irrespective of testosterone concentration. Contrary to this, testosterone significantly (*P* < 0.01) inhibited proliferation (0.958 ± 0.01) by 5% in group-I females but stimulated (1.101 ± 0.03) by 10% in group-III post pubertal females.

 There was no significant effect of testosterone on lymphocyte proliferation index in cycling buffaloes. However, proliferation was comparatively low in lymphocytes from day 0 (1.049 ± 0.03) as compared to day 10 (1.104 ± 0.03) of estrous cycle when treated with testosterone. The testosterone in all its concentrations stimulated proliferation to the level of 10% (1.104 ± 0.04) at 10 ng/mL and 5% (1.049 ± 0.04) at 0.01 ng/mL concentration in cyclic buffaloes.

### 3.2. Nitric Oxide Levels in Culture Supernatant

**Figure 1:**
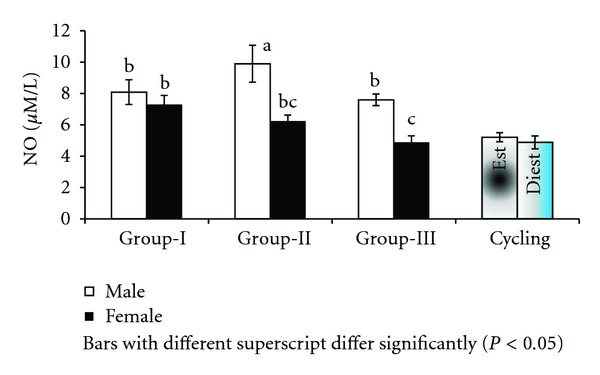
NO culture supernatant in Murrah buffaloes of different sex, age, and stage of cycle.

Nitric oxide was evaluated in supernatant collected 36 hours after lymphocyte culture (Figure 1). Males produced significantly (*P* < 0.001) higher (8.52 ± 0.39 *μ*M/L) NO in culture than females (6.13 ± 0.39 *μ*M/L). The difference was also significant (*P* < 0.05) among the three groups with lowest in group-III (6.23 ± 0.48 *μ*M/L), followed by group-II (8.07 ± 0.48 *μ*M/L) and group-I (7.68 ± 0.48 *μ*M/L). Among all groups, group-II male buffaloes registered the highest (9.89 ± 1.18 *μ*M/L) and group-III female buffaloes the lowest (4.87 ± 0.42 *μ*M/L) levels of nitric oxide in culture supernatant. In cyclic buffaloes the nitric oxide concentration in culture supernatant did not differ significantly with the stage of estrous cycle and was 5.20 ± 0.29 and 4.88 ± 0.41 *μ*M/L on day 0 (estrus) and day 10 (diestrus), respectively (Figure 1).

**Figure 2:**
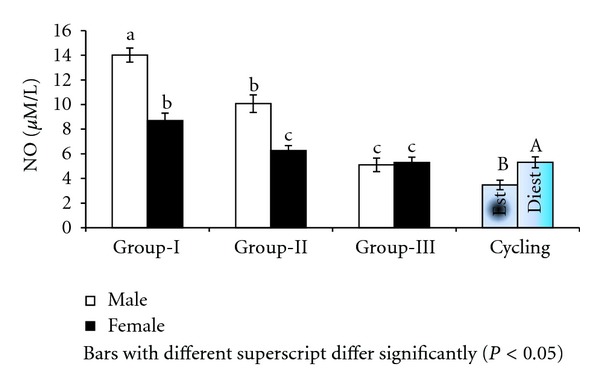
NO in culture supernatant supplemented with estradiol in Murrah buffaloes of different sex, age, and stage of cycle.

**Figure 3:**
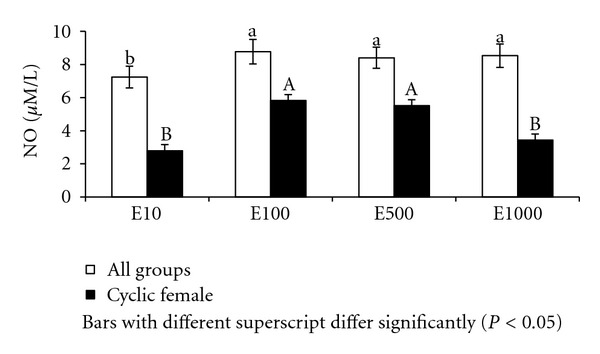
NO in culture supernatant supplemented with different levels of estradiol in Murrah buffaloes.

 Significantly (*P* < 0.001) higher levels of nitric oxide were recorded in culture supernatant of males (9.73 ± 0.30 *μ*M/L) than females (6.75 ± 0.30 *μ*M/L) after 36 hours of culture supplemented with estradiol. The NO levels in culture supernatants supplemented with estradiol were significantly (*P* < 0.001) different between age groups with group-I buffalo lymphocytes registering NO as high as 11.36 ± 0.37 *μ*M/L followed by group-II and group-III (Figure 2). Among the groups lymphocytes from group-I males registered highest NO (14.01 *μ*M/L) in culture supernatant (Figure 2). There was significant (*P* < 0.05) effect of different concentrations of estrogen on nitric oxide levels in culture supernatant; however, NO levels were maximum (8.77 ± 0.74 *μ*M/L) at 100 pg/mL concentration of estrogen and minimum at 10 pg/mL level (Figure 3).

In cyclic buffaloes, NO in culture supernatant of day 10 (diestrous) lymphocytes was (5.30 ± 0.44 *μ*M/L) significantly (*P* < 0.001) higher than (3.47 ± 0.38 *μ*M/L) day 0 (estrous) lymphocytes. Similarly different concentrations of estrogen in culture system had significant (*P* < 0.001) effect on NO in culture supernatant in cyclic buffaloes. Significantly higher levels of NO (5.82 ± 0.39 *μ*M/L and 5.51 ± 0.39 *μ*M/L) were recorded at 100 pg/mL and 500 pg/mL estradiol, respectively, as compared to (3.43 ± 0.93 and 2.79 ± 0.39 *μ*M/L) at 1000 pg/mL and 10 pg/mL estradiol, respectively, in cyclic buffaloes (Figure 3).

**Figure 4:**
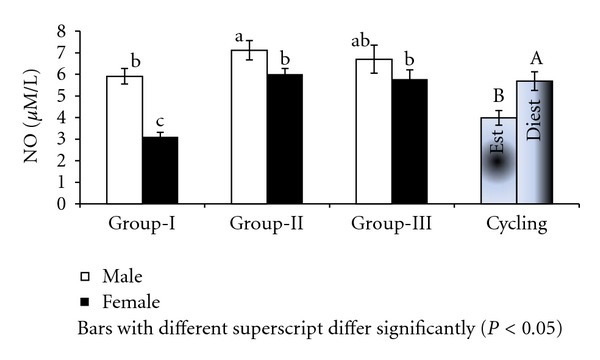
NO in culture supernatant supplemented with progesterone in Murrah buffaloes.

**Figure 5:**
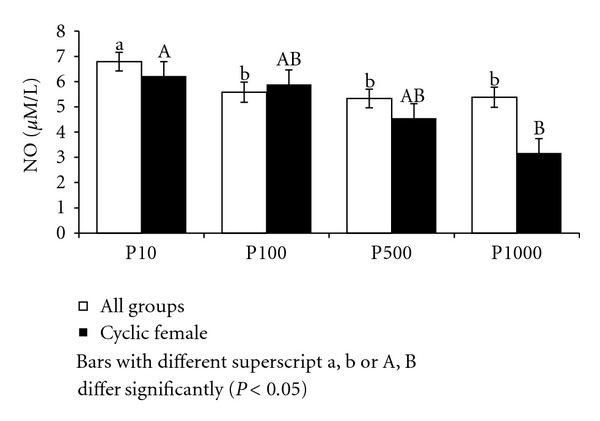
NO in culture supernatant supplemented with different levels of progesterone in Murrah buffaloes.

 Significant (*P* < 0.001) difference in levels of nitric oxide in culture supernatant supplemented with different concentrations of progesterone was registered between the sexes (6.58 ± 0.23 and 4.97 ± 0.23 *μ*M/L in males and females resp.) and between the age groups (4.50 ± 0.28, 6.56 ± 0.28, and 6.24 ± 0.28 *μ*M/L in group-I, group-II, & group-III buffaloes, resp.). Among the groups, group-II males registered highest (*P* < 0.05) levels of NO in culture supernatant where as group-I females the lowest (Figure 4). Similarly nitric oxide levels in culture supernatant differed significantly (*P* < 0.01) between various concentrations of progesterone treatment (Figure 5).

In cyclic buffaloes, phases of cycle influenced significantly the NO production in culture system. Lymphocytes from day 10 registered significantly (*P* < 0.01) higher (5.68 ± 0.40 *μ*M/L) NO in culture supernatant as compared to day 0 (3.98 ± 0.40 *μ*M/L) (Figure 4). There was significant (*P* < 0.01) decline in NO production with increasing concentrations of progesterone supplementation with highest NO at 10 ng/mL progesterone followed by 100, 500, and 1000 ng/mL progesterone respectively in cyclic buffaloes (Figure 5).

**Figure 6:**
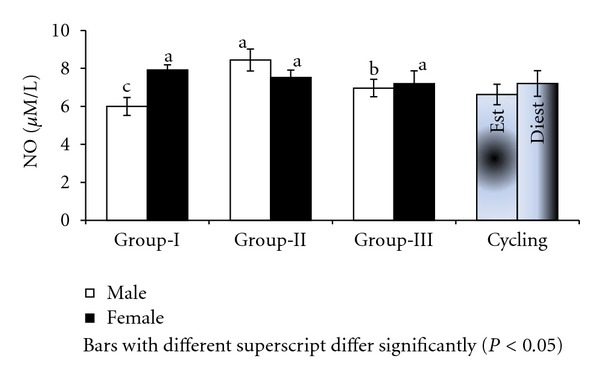
NO in culture supernatant supplemented with testosterone in Murrah buffaloes of different sex, age, and stage of cycle.

**Figure 7:**
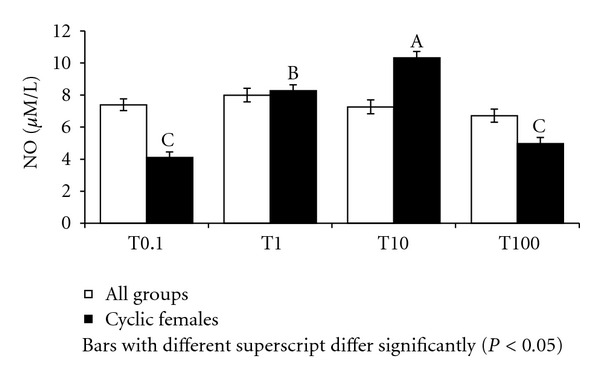
NO in culture supernatant supplemented with different levels of testosterone in Murrah buffaloes.

Analysis of variance revealed significant influence of sex on nitric oxide production in culture supernatant supplemented with testosterone and male and female buffaloes on an average, which produced 7.13 ± 0.23 and 7.54 ± 0.23 *μ*M/L NO, respectively. Further, NO in culture supernatant was significantly (*P* < 0.05) high (7.98 ± 0.29 *μ*M/L) in group-II buffaloes as compared to group-III (7.08 ± 0.29) and group-I (6.96 ± 0.29 *μ*M/L). Among the all groups, group-II male buffaloes recorded the highest (*P* < 0.01) NO whereas group-I males the lowest (Figure 6). The levels did not vary significantly in culture supernatant in response to different levels of testosterone (Figure 7). However, the levels were comparatively higher (7.99 ± 0.41 *μ*M/L) with 10 ng/mL supplementation.

 In cyclic buffaloes, the estrous and diestrous phases of reproductive cycle did not significantly influence NO production in culture supplemented with different concentrations of testosterone and mean levels of NO in supernatant were 7.20 ± 0.35 and 6.62 ± 0.35 *μ*M/L in day 10 and day 0 lymphocyte culture, respectively (Figure 6). However, among the testosterone concentrations, NO production at 0.1 and 100 ng/mL concentrations was similar and significantly lower than at 1 ng/mL and 10 ng/mL (Figure 7).

## 4. Discussion

 In vitro lymphocyte proliferation was measured in response to supplementation of different doses of estrogen in culture. Estrogen inhibited proliferation slightly in lymphocytes of group-I and group-II but stimulated in group-III buffaloes of either sex. However, among four concentrations of estradiol, 100 pg/mL was stimulatory in buffaloes of all ages of either sex as compared to 1000 and 10 pg/mL. These findings reflect the dose-dependent effect of estrogen on lymphocyte proliferation. Possibly certain levels of estrogen are immuno-stimulatory and enhance cell mediated immunity but at the same time very high doses are immunosuppressive. An interesting finding was that, in post pubertal females, estrogen at higher concentration also had stimulatory effect on lymphocyte proliferation. There is no consistency in available reports on lymphocyte proliferation delineating similar type of response in relation to estrogen in different animals. Marco et al. [19] reported inhibitory effects of estradiol on lymphocyte proliferation in both males and females at 50 nM concentration that upholds the present findings. Athreya et al. [20] used 0.3 to 3 ng/mL of estradiol and reported no consistent effects on human PBMCs proliferation in vitro. Similarly Holdstock et al. [21] used estradiol at 40 ng/mL in culture and reported no significant effect of it on mitogen-induced proliferation in humans. Bilbo and Nelson [12] reported stimulatory effect of estrogen at all levels on lymphocyte proliferation in vitro in hamsters, contrary to many other studies. All these findings suggest the charisma of species variation in the effects of estradiol on cell-mediated immunity. Further, estrogen had different effects on lymphocyte proliferation from estrous and diestrous stage of reproductive cycle with significantly higher stimulation in diestrous phase lymphocytes than estrous. Since group-III females in present study were actually progesterone dominated therefore, to counteract the negative effects of immunosuppressive progesterone, estrogen even at higher concentrations actually might have boosted the blastogenic activity of lymphocytes in diestrous cyclic buffaloes rather than inhibiting as was true for other nonprogesterone phases like estrous. These present results are comparable to the findings of Sugiura et al. [22] who reported significantly enhanced response of dog PBMNCs collected in anestrus to PYO-252 upon the addition of estradiol-17 beta to the culture. There are reports of presence of steroid hormone receptors in immune cells [23–27] and presence or absence, upregulation or downregulation of receptors may be the probable reason for the varied responses of lymphocytes to estrogen treatment. There is possibility of different outcome of genomic and nongenomic pathway of steroid actions on lymphocyte blastogenesis and depending upon the receptor concentration stimulation or inhibition of lymphocyte proliferation may be regulated. Therefore, it seems important to investigate the receptor expression in immune cells at different proliferation responses to arrive at a comprehensible conclusion.

 Progesterone supplementation at different concentrations in all buffalo groups of either sex inhibited proliferation with more severe inhibition in males (10%) as compared to females (3%). This sex-dependent variability in progesterone effects may probably be due to the adaptation of lymphocytes to progesterone because of being exposed to it in females. There was an inverse relationship between the dose and proliferation index. The highest concentration of progesterone (1000 ng/mL) inhibited proliferation by 19% whereas the lowest (10 ng/mL) registered stimulatory effect of 6%. These findings are in partial agreement with those reported by Marco et al. [19] in mice who reported inhibitory effect of progesterone only in females. There are several reports which indicate immunosuppressive effects of progesterone. R. Druckmann and M. A. Druckmann [28] reported that progesterone up regulated progesterone receptors on lymphocytes in placenta and was responsible for production of progesterone-induced blocking factor which favours conception and disfavours the rejection of conceptus. Su et al. [29] reported inhibitory effects of progesterone on immune response to lipopolysaccharide (LPS) through modulating Toll-like receptor (TLR) signalling. Wulster-Radcliffe et al. [9] in gilts and Kaushic et al. [6] in rodents reported that exogenous progesterone increased the risk of development of diseases indicating inhibitory effects of progesterone on cell-mediated immune responses. Sugiura et al. [22] examined the proliferative response of PBMNCs to PYO-252 and reported significantly decreased response in the first half (day 10) of estrous cycle, but increased in proestrous/estrous and these responses were significantly suppressed in the presence of progesterone. All these reports suggest the immunosuppressive effects of progesterone and in principle confirm the present finding in buffaloes with progesterone having inhibitory effect on cell-mediated immunity.

In current investigation, testosterone treatment stimulated lymphocyte proliferation in both males and females to the tune of 2 and 5%, respectively, with no significant dose-dependent effect. Testosterone treatment in lymphocyte culture did not yield any dependable and lucid effects either at any particular dose or in any age group. In group-I males it stimulated proliferation at all concentrations of testosterone with maximum at 0.1 ng/mL but in case of group-I female it inhibited proliferation at all concentrations of testosterone. In group-II males contrary to group-I males, testosterone inhibited proliferation except at 1 ng/mL concentration but at the same time in group-II females it stimulated proliferation at all concentrations of testosterone with maximum at 1 ng/mL. Whereas in group-III post pubertal males picture was same as that of group-II males but in group-III females, testosterone stimulated proliferation at all concentrations with maximum at 100 ng/mL. With these varied effects of testosterone, it is hard to arrive at any definitive conclusion. It may be possible that the lymphocytes possess the capability to convert testosterone into estrogen, which may govern the proliferation in adult buffaloes. This option may be working since we got similar observations with testosterone and estrogen in post pubertal female lymphocytes. Benten et al. [30] have demonstrated a membrane testosterone receptor on lymphocyte enabling T cells a nongenomic response to testosterone. Therefore, research in steroid receptors and converting enzymes in lymphocytes is warranted. Bilbo and Nelson [12] in hamsters found that both testosterone as well as estradiol is stimulatory to lymphocyte proliferation, whereas Marco et al. [19] in mice reported no significant effect of testosterone on lymphocyte proliferation or cytokine secretion. Roden et al. [13] reported improved immune responses including lymphocyte proliferation on deprivation of testosterone in rodent model. Duffy et al. [31] in wild male and female starling birds reported that implant of testosterone significantly reduced humoral immunity in both males and females. All these reports indicate species differences in testosterone effects on immune responses. Present findings revealed testosterone as stimulatory to lymphocyte proliferation in buffaloes of all ages and sexes at least at 1 ng/mL concentration. Although, current knowledge of sex steroid hormone effects on immune function is relatively limited to that of mice, rats, and humans [32, 33], the results of the current study in buffaloes suggest a unique role for the sex steroids in influencing immune functions that may have evolved along with the distinct natural history of these animals.

### 4.1. Nitric Oxide in Culture Supernatant Cultured with Sex Steroids

 Sex and age bias in NO production by lymphocytes in culture was evident in present study. Significantly high levels of NO were found in culture supernatant of males as compared to females treated with estrogen. Among the groups, group-I buffaloes had more NO in culture as compared to group-II and III. However, production rate was not affected by increasing dose of estrogen in culture. The proliferation was higher in group-III where NO was lowest suggesting inverse relationship between PI and nitric oxide production in culture and probably estrogen affected proliferation through NO levels in lymphocyte culture. However, the NO in culture supernatant of day 10 lymphocytes was significantly higher than day 0 while proliferation was also significantly high at diestrous than estrous phase. NO has been reported to be secreted by lymphocytes [34, 35] and blood leukocytes [36] in cattle. NO is liberated either spontaneously or after cleavage by ectoenzymes found on T and B lymphocytes in bovines [35, 37].

 Nitric oxide in lymphocyte culture treated with different concentrations of progesterone was significantly high in males as compared to females. Among the groups, significantly high levels of NO in culture supernatant were found in group-III and least in group-I buffaloes. In cyclic buffaloes, diestrous phase lymphocytes produced more NO than estrous phase, treated with progesterone. Similarly the nitric oxide levels in supernatant differed between the concentrations of progesterone with lowest NO at highest progesterone concentration. The same trend was also observed in lymphocyte proliferation. This suggested that progesterone probably has no direct effect on NO production by lymphocytes and as such more proliferation is accompanied with more NO.

 NO in lymphocyte culture supernatant at any level of testosterone did not show any significant difference between sexes with male and female buffaloes on an average, producing 7.13 ± 0.23 and 7.54 ± 0.23 *μ*M/L NO, respectively, in culture. This indicated that the lymphocytes obtained from female animals were equally responsive to testosterone as males. However, levels tended to be significantly (*P* < 0.05) high in group-II buffaloes as compared to group-I and III. In general, low levels of proliferation were associated with less production of NO in culture in different age groups of buffaloes. Differences were not significant during different stages of estrous cycle which suggested that variations in the immune responses during estrous cycle were not related to testosterone as was evident through PI also.

Since it happens to be the first report to register NO in culture supernatant of lymphocytes from buffaloes of different sex and age with sex steroid treatment, it needs further investigation before arriving at any final conclusion on relationship of sex steroids, NO, and lymphocyte proliferation.

## 5. Conclusion

Effect of female sex steroids on lymphocyte proliferation in vitro was found to be existing. Estradiol effects were dose dependent too, and high as well as too low levels of estradiol were inhibitory to lymphocyte proliferation. Progesterone was found to be inhibiting lymphocyte proliferation and there was inverse relationship between progesterone dose and lymphocyte proliferation. Testosterone did not show any consistent and lucid effects on lymphocyte proliferation, nor did it inhibit proliferation at any dose level. The proliferation was higher in group-III where NO was lowest suggesting inverse relationship between PI and nitric oxide production in culture and probably estrogen affected proliferation through NO levels in lymphocyte culture. NO was not the candidate mediator of effects of all sex steroids in lymphocyte proliferation. NO levels in supernatant differed between the concentrations of progesterone with lowest NO at highest progesterone concentration and lowest proliferation. This suggested that progesterone probably has no direct effect on NO production by lymphocytes and as such more proliferation is accompanied with more NO. No significant difference in NO culture supernatant was observed at different concentrations of estradiol or testosterone.

## Figures and Tables

**Table 1:** Dose-dependent effect of estradiol 17*β* on lymphocyte proliferation in vitro in Murrah buffaloes of different sex and age.

Age group	Sex	Estradiol 17*β* concentration (pg/mL)				Sex* group mean	Group mean	Sex mean
		10	100	500	1000			
Group-I	Male	0.98 ± 0.03	1.04 ± 0.04	0.93 ± 0.04	0.87 ± 0.04	0.95 ± 0.02	0.95^B^ ± 0.01	0.97 ± 0.01 (Male)
	Female	0.93 ± 0.03	0.99 ± 0.02	0.93 ± 0.02	0.93 ± 0.02	0.94 ± 0.01		
Group-II	Male	0.95 ± 0.04	1.01 ± 0.05	0.97 ± 0.04	0.91 ± 0.01	0.96 ± 0.02	0.95^B^ ± 0.01	
	Female	0.93 ± 0.04	1.01 ± 0.03	0.96 ± 0.02	0.83 ± 0.02	0.93 ± 0.02		0.99 ± 0.01 (Female)
Group-III	Male	0.91 ± 0.02	1.12 ± 0.06	1.07 ± 0.05	0.86 ± 0.05	0.99 ± 0.03	1.04^A^ ± 0.01	
	Female	0.99 ± 0.02	1.11 ± 0.08	1.14 ± 0.08	1.14 ± 0.08	1.09 ± 0.03		
Treatment mean		0.95^b^ ± 0.01	1.05^a^ ± 0.02	1.00^a^± 0.02	0.93^b^ ± 0.02	

Values with superscript a, b within same row and A, B within same column differ significantly (*P* < 0.05). CD at *t* 5%: sex: 0.03, group: 0.04, treatment: 0.05, sex* group: 0.06 and sex*group*treatment: 0.129.

**Table 2:** Dose dependent effect of progesterone on lymphocyte proliferation in vitro in Murrah buffaloes of different sex and age.

Age group	Sex	Progesterone concentration (ng/mL)				Sex* group mean	Group mean	Sex mean
		10	100	500	1000			
Group-I	Male	0.98 ± 0.03	0.90 ± 0.03	0.92 ± 0.02	0.86 ± 0.01	0.91 ± 0.01	0.92 ± 0.01	0.90 ± 0.01 (Male)
	Female	0.99 ± 0.07	0.96 ±0.04	0.93 ± 0.02	0.83 ± 0.04	0.92 ± 0.02		
Group-II	Male	1.09 ± 0.07	1.07 ±0.05	0.87 ± 0.03	0.73 ± 0.02	0.94 ± 0.03	0.94 ± 0.01	
	Female	1.01 ± 0.01	1.00 ± 0.04	0.89 ± 0.02	0.76 ± 0.01	0.94 ± 0.02		0.97 ± 0.01 (Female)
Group-III	Male	1.06 ± 0.05	0.89 ± 0.04	0.77 ± 0.04	0.71 ± 0.05	0.86 ± 0.03	0.96 ± 0.01	
	Female	1.14 ± 0.07	1.07 ± 0.05	1.05 ± 0.05	0.99 ± 0.03	1.06 ± 0.02		
Treatment mean		1.06^a^ ± 0.02	0.98^b^ ± 0.02	0.90^c^ ± 0.02	0.81^d^ ± 0.02	

Values with different superscript within same row differ significantly (*P* < 0.05). CD at *t* 5%: sex: 0.03, treatment: 0.05, sex* group: 0.06 and sex* group* treatment: 0.123.

**Table 3:** Dose-dependent effect of testosterone on lymphocyte proliferation in vitro in buffaloes of different sex and age.

Age group	Sex	Testosterone concentration (ng/mL)				Sex* group mean	Group mean	Sex mean
		0.10	1	10	100			
Group-I	Male	1.27 ± 0.06	1.04 ± 0.04	1.18 ± 0.04	1.02 ± 0.04	1.13 ± 0.03	1.04 ± 0.017	1.03 ± 0.01 (Male)
	Female	0.97 ± 0.03	0.94 ± 0.02	0.94 ± 0.02	0.98 ± 0.03	0.96 ± 0.01		
Group-II	Male	0.95 ± 0.03	1.05 ± 0.03	0.93 ± 0.04	0.91 ± 0.03	0.96 ± 0.02	1.03 ± 0.017	
	Female	1.11 ± 0.05	1.140 ± 0.04	1.08 ± 0.05	1.07 ± 0.03	1.10 ± 0.02		1.05 ± 0.01 (Female)
Group-III	Male	0.94 ± 0.05	1.05 ± 0.05	1.06 ± 0.03	0.90 ± 0.03	0.99 ± 0.02	1.04 ± 0.017	
	Female	1.06 ± 0.06	1.08 ± 0.05	1.12 ± 0.05	1.14 ± 0.09	1.10 ± 0.03		
Treatment mean		1.05 ± 0.02	1.05 ± 0.02	1.05 ± 0.02	1.00 ± 0.02	

CD at* t* 5%: sex: 0.03, group: 0.04, sex* group: 0.06, sex* group* treatment: 0.13.
